# Diagnostic Journey From Suspected Ovarian Mass to Rare Mesothelial Cyst

**DOI:** 10.7759/cureus.72505

**Published:** 2024-10-27

**Authors:** Giorgiana Franzese, Danny J Koh

**Affiliations:** 1 Obstetrics and Gynecology, Midwestern University Chicago College of Osteopathic Medicine, Downers Grove, USA; 2 Obstetrics and Gynecology, Northwestern University Feinberg School of Medicine, Geneva, USA

**Keywords:** benign cystic mesothelioma, benign mesothelial cyst, mesothelial cyst, pelvic cystic mass, pelvic mass

## Abstract

Mesothelial cysts are rare, benign formations originating from the mesothelial cells lining body cavities. These cysts are more prevalent in women of reproductive age but can also be found in men and children. We present the case of a 38-year-old woman with a large pelvic mass initially suspected to be ovarian in origin. Imaging studies revealed complex cystic structures, and surgical exploration was necessary for a definitive diagnosis. Pathological examination confirmed a benign mesothelial-lined cyst. Despite their rarity and benign nature, mesothelial cysts should be included in the differential diagnosis of pelvic cysts. Recognizing these lesions can help prevent misdiagnosis and ensure appropriate management and counseling. More research needs to be done, especially regarding any implication to fertility potential.

## Introduction

Mesothelial cysts are rare, benign cysts arising from mesothelial cells that line the body’s cavities. Such cysts have been found in various areas of the abdominal peritoneal surfaces, more commonly around the round ligament, mesentery, and omentum, and less commonly around the uterus [1,2]. These cysts are most commonly found in women of reproductive age, though they can also occur in children and men. To date, fewer than 200 cases have been reported or described in the literature, with most occurring in reproductive-aged women [3]. The exact etiology remains uncertain, but it is widely believed to involve a chronic inflammatory process similar to that seen in endometriosis [3]. When diagnosing pelvic lesions, it is important to consider the possibility of uterine mesothelial cysts. Raising awareness of this uncommon condition can enhance evaluation, decision-making, and overall management of the disease.

## Case presentation

A 38-year-old female, gravida 3, para 2-0-1-2, initially presented to her primary care physician with persistent left lower quadrant pain. After further evaluation, a computed tomography scan of the abdomen and pelvis (CTAP) revealed an adnexal cyst, leading to her referral to the gynecology clinic for further management. The patient reported bladder pain and frequency without dysuria. The pain was slightly alleviated at rest but persisted during urination. These symptoms had been ongoing for two weeks, with no prior history of similar issues. She did not experience significant pain during intercourse, although certain positions seemed to exacerbate the discomfort. Additionally, she noted mid-pelvic pain associated with various body position changes. The patient had no relevant medical history aside from a Mirena intrauterine device (IUD) that was confirmed in place. Clinically, the abdomen was soft and non-distended but tender to palpation suprapubically. A pelvic exam revealed bilateral adnexal fullness and a palpable mass near the mid-pubic region, which caused the patient discomfort. Subsequent lab tests, including CEA, CA-125, CA 19-9, Alpha-Fetoprotein (AFP), beta-HCG, and Inhibin A/B, were all within normal limits.

**Figure 1:**
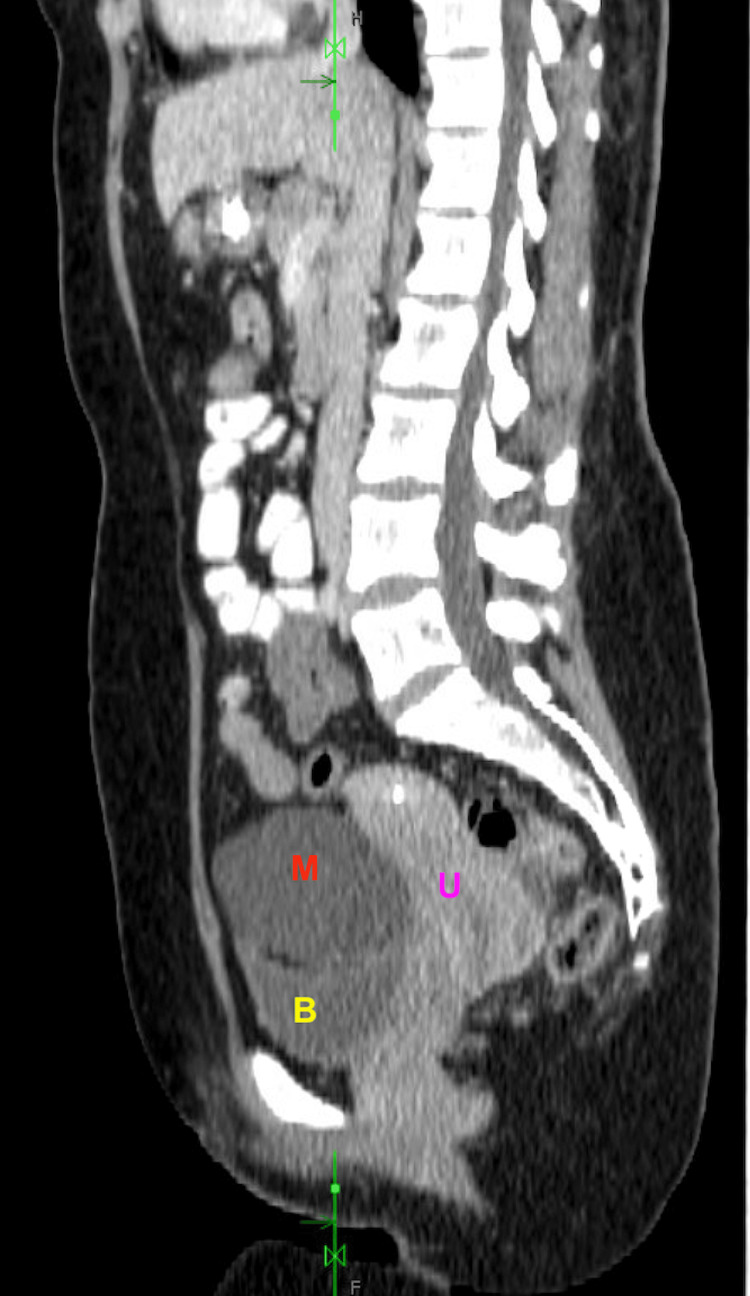
CT abdomen and pelvis demonstrating mesothelial cyst Labels: M represents the mesothelial cyst, B denotes the bladder, and U indicates the uterus

**Figure 2:**
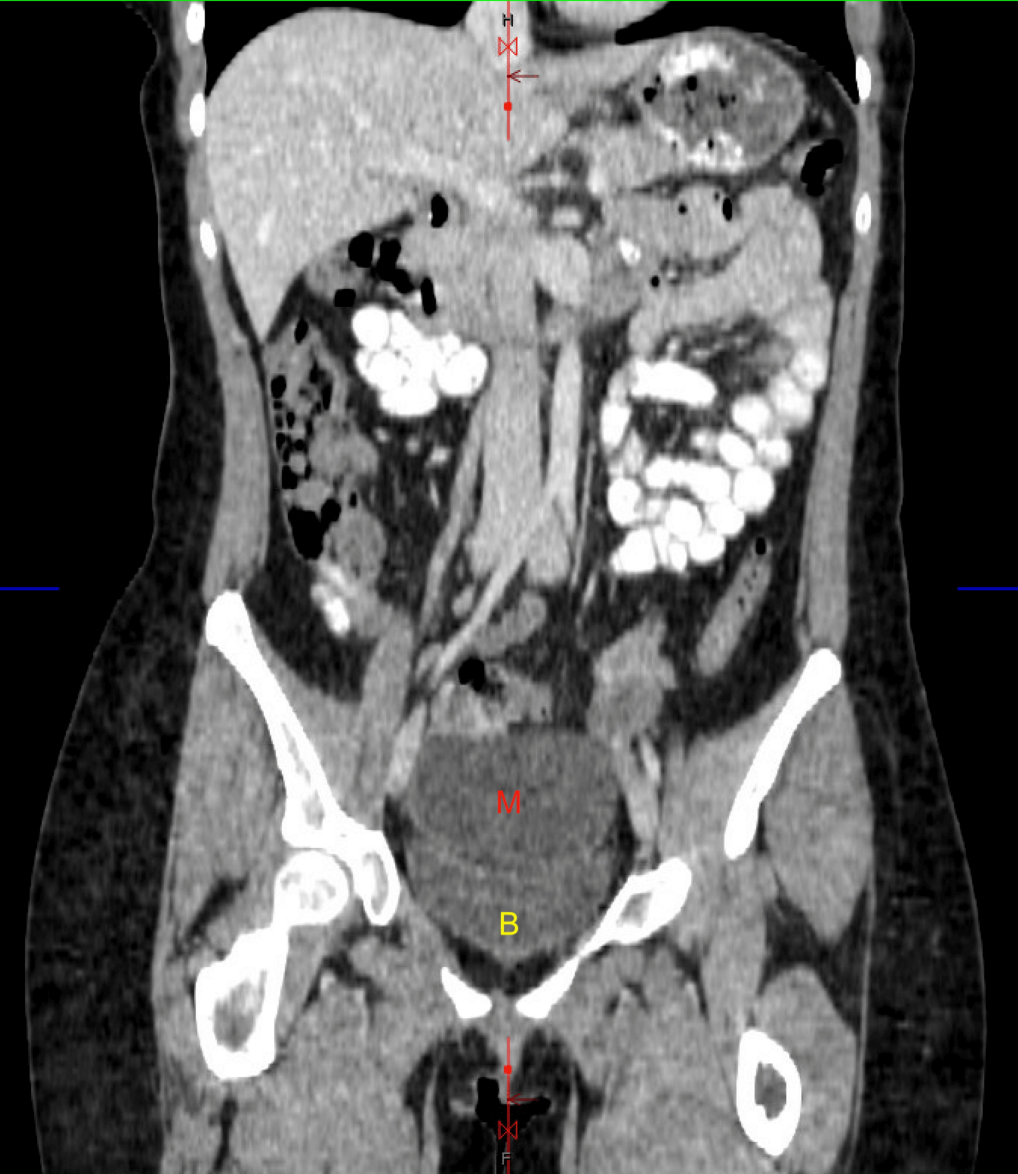
Coronal CT scan of the pelvis demonstrating a pelvic mass (M) exerting mass effect on the bladder (B)

A CTAP with contrast revealed an oval-shaped, low-density lesion within the central anterior pelvis, measuring approximately 8.5 cm, suspected to originate from the right adnexa as seen in Figure 1 and Figure 2.

**Figure 3:**
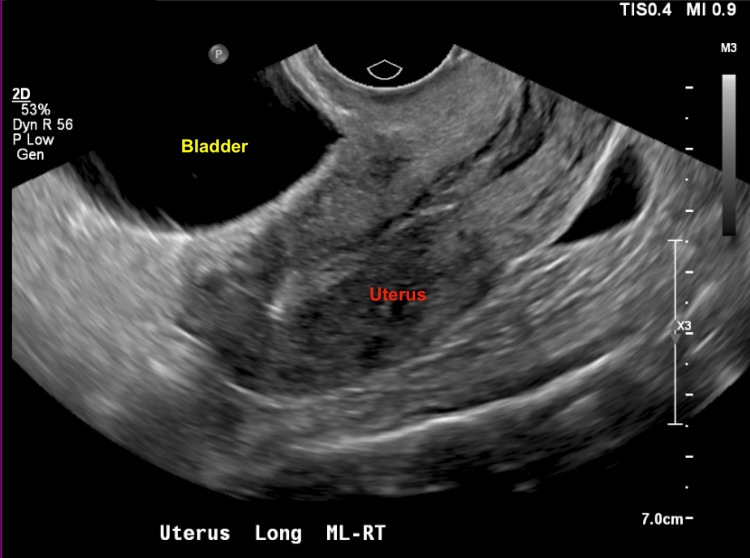
Transvaginal ultrasound demonstrating the uterus and bladder No visible mesothelial cyst is noted in this view, contrasting with the presence of the mesothelial cyst seen in the corresponding CT scan.

The lesion created a significant mass effect on the bladder, as demonstrated in Figure 1 and Figure 2. However, a subsequent pelvic ultrasound did not reveal the 8.5 cm cyst that had been clearly identified on the CT scan, as shown in Figure 3.

**Figure 4:**
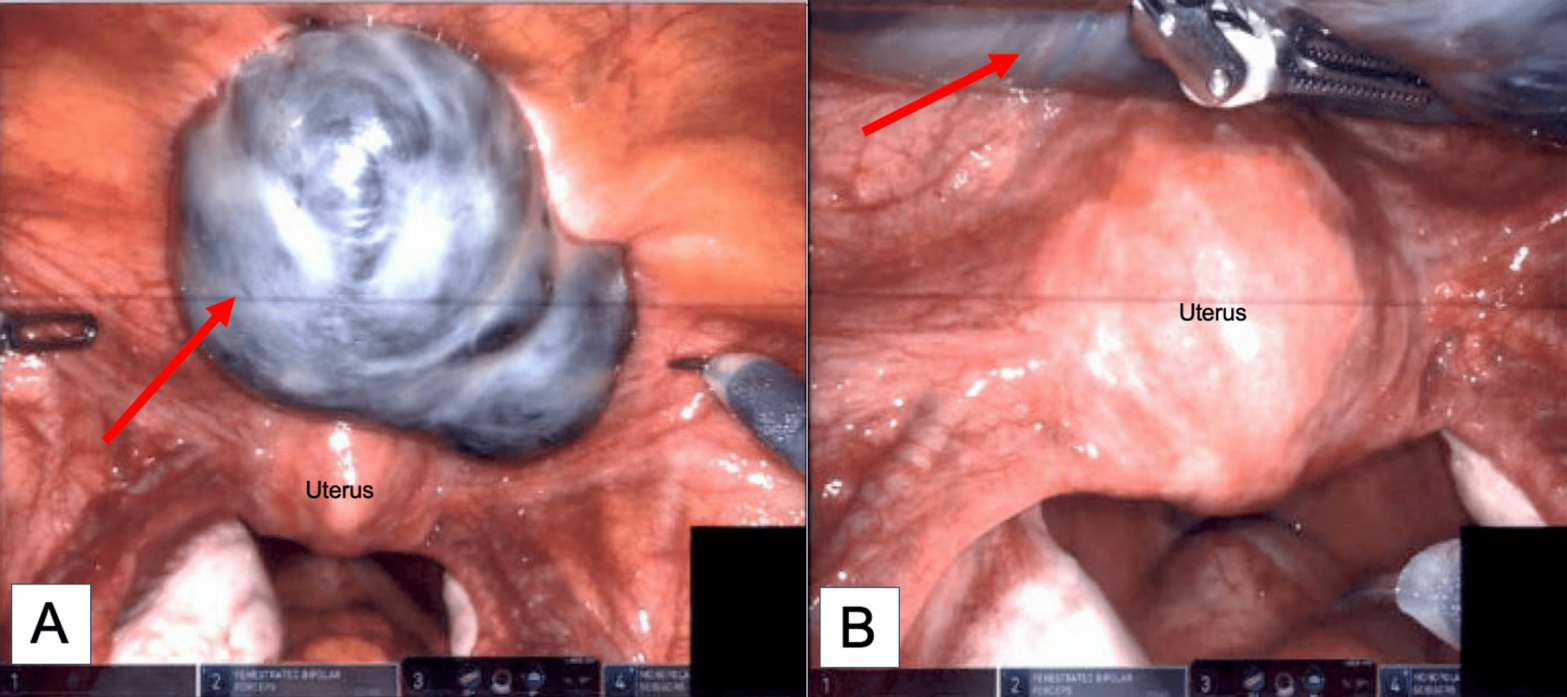
Laparoscopic images of an 8.5 cm mesothelial cyst located on the anterior pelvic wall A) Red arrow pointing to the mesothelial cyst. B) The mesothelial cyst is seen anterior to the uterus, embedded in the anterior pelvic wall.

A robotic-assisted laparoscopic cystectomy was performed. Intraoperative findings included an 8.5 cm cyst on the anterior pelvic wall, superficial to the bladder serosa, suspected to be a satellite endometrial cyst, as seen in Figure 4. Endometriosis implants were also noted in the pelvis, although no images were taken of these.

**Figure 5:**
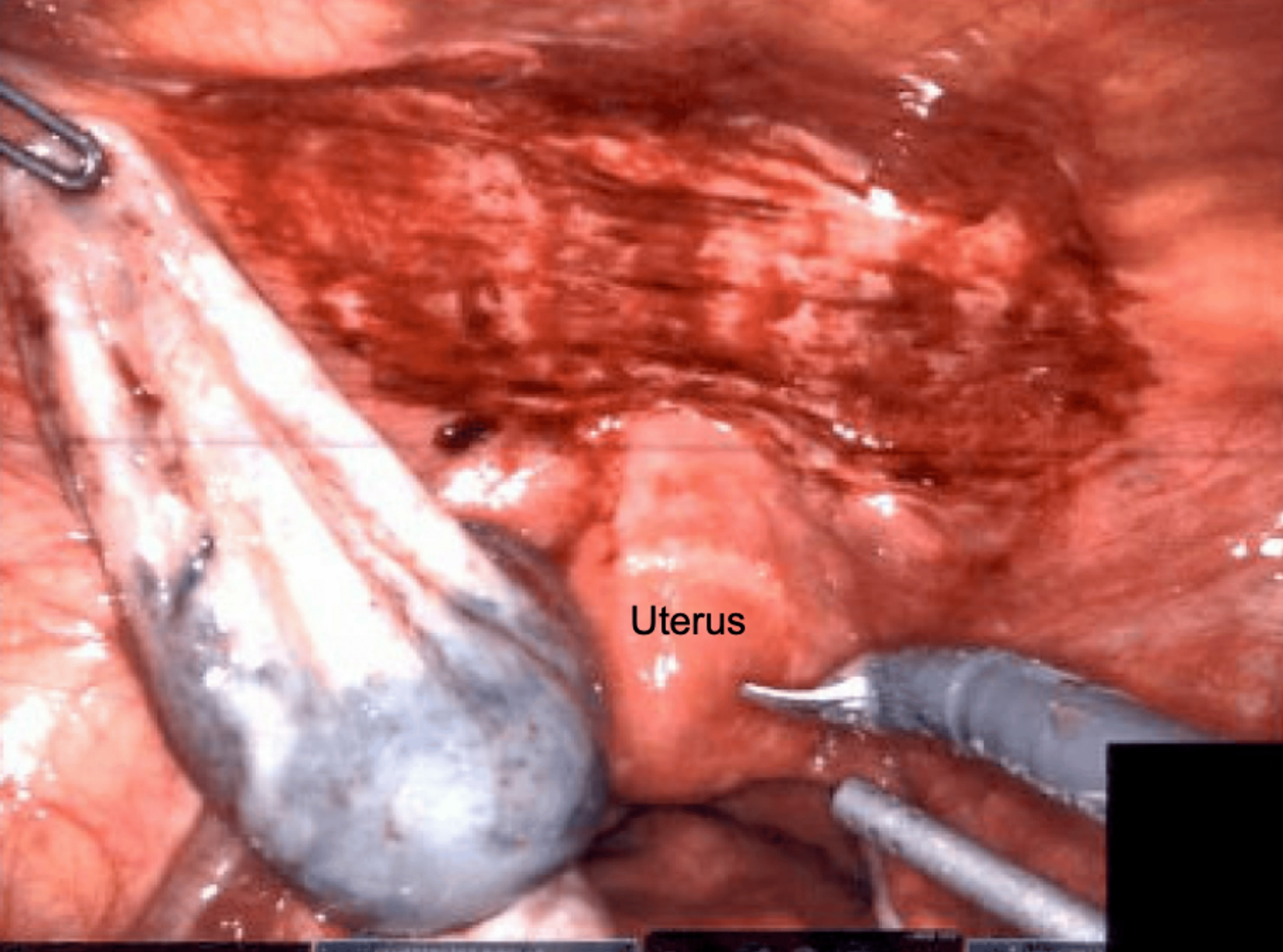
Intraoperative image showing the cyst remnant held in the grasping forceps after removal The image highlights the clean surgical field and careful dissection to avoid damage to surrounding structures such as the uterus and bladder. Minimal bleeding is observed, demonstrating effective hemostasis.

The pelvic wall cyst was carefully dissected and completely removed without any complications as shown in Figure 5.

All specimens were sent for routine pathological examination and confirmation of diagnosis. Pathohistological and immunohistochemical examinations confirmed the diagnosis of a benign mesothelial-lined cyst of the pelvic wall.

Outcome and follow-up

The patient recovered appropriately following robotic-assisted laparoscopic resection of the 8.5 cm satellite mesothelial cyst above the bladder serosa. The patient was followed up at four weeks postoperatively. The urinary function has normalized with no pain during urination, though mild discomfort persists when emptying the bladder. 

Continued surveillance includes routine monitoring for signs of recurrence, with emphasis on recognizing symptoms such as pelvic pain, abdominal bloating, urinary changes (frequency or difficulty), menstrual irregularities, and gastrointestinal discomfort. The patient has been advised to closely monitor her symptoms and to report any immediately for reassessment.

## Discussion

Uterine tumors or cysts are often associated with ovarian cysts, cystic degeneration of leiomyomas, endometriomas, serosal inclusion cysts, and rarely, benign mesothelial-lined cysts [1]. Despite their rarity and benign nature, mesothelial cysts should be included in the differential diagnosis of pelvic masses. Due to their uncommon occurrence, these cysts are frequently misdiagnosed as ovarian cysts either preoperatively or postoperatively [4]. In our case, the patient was initially suspected to have a satellite endometrial cyst.

Mesothelial cysts originate from mesothelial cells, which form the epithelium of serous membranes such as the peritoneum, pleura, and pericardial cavities. Alternative names for mesothelial cysts have been suggested, such as benign cystic mesothelioma, peritoneal inclusion cysts, inflammatory cysts in the peritoneum, and postoperative peritoneal cysts [4]. Mesothelial inclusion cysts are rare benign tumors, with fewer than 200 cases reported in the literature [3]. These cysts are typically found in the pelvic and abdominal regions and can occur in both men and women, though they are more common in women of reproductive age [5]. Mesothelial cysts can be asymptomatic, especially when small, but larger cysts most commonly cause abdominal pain, discomfort, or gastrointestinal obstruction, necessitating surgical intervention.

The pathogenesis of mesothelial cysts is not well understood, but it is widely believed to involve a chronic inflammatory process similar to endometriosis [3]. Common symptoms associated with mesothelial cysts include increasing abdominal girth, abdominal pain, nausea, vomiting, and constipation [3]. Diagnosis often involves imaging techniques such as ultrasound, CT scans, or magnetic resonance imaging (MRI). However, mesothelial cysts can mimic other conditions like ovarian cancers and cystic lymphangiomas on imaging, complicating preoperative diagnosis [6]. Biopsy and histopathological examination, including the use of immunohistochemical stains specific to mesothelial cells such as calretinin and cytokeratin 5/6, are essential for a definitive diagnosis [1,7]

In this case report, the patient's ultrasonography was unable to visualize the mesothelial cyst. Generally on ultrasound, mesothelial cysts appear as cystic masses without calcifications [3]. MRI and CT imaging typically reveal multilocular or unilocular cystic lesions with thin-walled septations and fluid densities [7]. Mesothelial cysts have a high recurrence rate, with an average interval of 32 months, and therefore require ongoing monitoring [3,8]. While rare, there have been reported cases of malignant transformation into diffuse malignant mesotheliomas [9].

## Conclusions

Despite their rarity and benign nature, mesothelial cysts should be considered in the differential diagnosis of pelvic cysts to prevent misdiagnosis and ensure appropriate management and counseling. Although complete excision of the cyst wall during surgery can be challenging, the overall prognosis for patients is generally favorable. However, there remains a critical need for further research, particularly to explore the potential impact of mesothelial cysts on fertility.
